# Transcriptomes Suggest That Pinniped and Cetacean Brains Have a High Capacity for Aerobic Metabolism While Reducing Energy-Intensive Processes Such as Synaptic Transmission

**DOI:** 10.3389/fnmol.2022.877349

**Published:** 2022-05-09

**Authors:** Cornelia Geßner, Alena Krüger, Lars P. Folkow, Wilfrid Fehrle, Bjarni Mikkelsen, Thorsten Burmester

**Affiliations:** ^1^Institute of Zoology, University of Hamburg, Hamburg, Germany; ^2^Department of Arctic and Marine Biology, UiT The Arctic University of Norway, Tromsø, Norway; ^3^Institute of Pathology With the Sections Molecular Pathology and Cytopathology, University Medical Center Hamburg-Eppendorf, Hamburg, Germany; ^4^Faroe Marine Research Institute, Tórshavn, Faroe Islands

**Keywords:** hooded seal, neurons, hypoxia, transcriptome, marine mammals, brain, diving, cetacean

## Abstract

The mammalian brain is characterized by high energy expenditure and small energy reserves, making it dependent on continuous vascular oxygen and nutritional supply. The brain is therefore extremely vulnerable to hypoxia. While neurons of most terrestrial mammals suffer from irreversible damage after only short periods of hypoxia, neurons of the deep-diving hooded seal (*Cystophora cristata*) show a remarkable hypoxia-tolerance. To identify the molecular mechanisms underlying the intrinsic hypoxia-tolerance, we excised neurons from the visual cortices of hooded seals and mice (*Mus musculus*) by laser capture microdissection. A comparison of the neuronal transcriptomes suggests that, compared to mice, hooded seal neurons are endowed with an enhanced aerobic metabolic capacity, a reduced synaptic transmission and an elevated antioxidant defense. Publicly available whole-tissue brain transcriptomes of the bowhead whale (*Balaena mysticetus*), long-finned pilot whale (*Globicephala melas*), minke whale (*Balaenoptera acutorostrata*) and killer whale (*Orcinus orca*), supplemented with 2 newly sequenced long-finned pilot whales, suggest that, compared to cattle (*Bos taurus*), the cetacean brain also displays elevated aerobic capacity and reduced synaptic transmission. We conclude that the brain energy balance of diving mammals is preserved during diving, due to reduced synaptic transmission that limits energy expenditure, while the elevated aerobic capacity allows efficient use of oxygen to restore energy balance during surfacing between dives.

## Introduction

The mammalian brain represents only ∼2% of body mass but requires ∼20% of the consumed oxygen at rest (Kety, 1957; Siesjö, 1978). The high energy expenditure is mainly explained by costs for maintenance of ion gradients, neuronal signal transmission and the cycling of neurotransmitters. These processes consume 80% of the ATP produced in neuronal mitochondria (Attwell and Laughlin, 2001; Shulman et al., 2004). To meet the high energy needs and because the brain has limited endogenous energy reserves, the brain depends on a constant vascular oxygen and substrate supply to fuel ATP production, making it the organ that, apart from the heart, is most vulnerable to hypoxia. Hypoxic conditions in the brain are observed in many neurological disorders such as stroke, trauma, hemorrhage and perinatal encephalopathy, as well as Alzheimer’s disease and Parkinson’s disease (Ogunshola and Antoniou, 2009).

The structure and function of the brain in general is well studied in standard model organisms. However, in species adapted to extreme conditions, such as recurrent hypoxia, the mechanisms to cope with these challenges are less well understood. Such species present a valuable opportunity to study molecular mechanisms that underlie hypoxia tolerance. Pinnipeds, for example, have evolved to cope with regular periods of limited oxygen availability during diving. Thus, diving mammals display a range of adaptations that allow them to withstand periods of severe hypoxia without damage to their brain (Kerem and Elsner, 1973; Butler and Jones, 1997; Ramirez et al., 2007; Meir et al., 2009; Blix, 2018), which enable deep-diving species, like the Weddell seal (*Leptonychotes weddelli*) and the northern elephant seal (*Mirounga angustirostris*), to cope with severe hypoxemia during diving and with arterial oxygen tensions routinely dropping below 20 mmHg (Qvist et al., 1986; Meir et al., 2009; Ponganis, 2019). The hooded seal (*Cystophora cristata*) can dive to depths of >1000 m and remain submerged for more than 52 min (Folkow and Blix, 1999; Vázquez-Medina et al., 2011). The neurons of the hooded seal were shown to have an intrinsic tolerance toward hypoxia when fresh brain slices were exposed to hypoxia *in vitro*. Neurons of the visual cortex and hippocampus survived experimental hypoxia at least 4–5 times longer than did mouse neurons. Further, the synaptic activity of hypoxia-exposed hooded seal neurons made an astonishing recovery to normoxic levels even after several hours of exposure to severe hypoxia (Folkow et al., 2008; Geiseler et al., 2016).

Previous transcriptome studies of nervous tissue in diving mammals utilized whole tissue samples (neurons and glia cells). Fabrizius et al. (2016) compared transcriptomes of the visual cortex of the hooded seal with those of the ferret, which suggested that the capacity for aerobic metabolism may be reduced in the hooded seal brain. Hoff et al. (2017) compared fresh slices of the hooded seal visual cortex that were exposed to hypoxia and oxidative stress *in vitro*, to slices exposed to normoxia, and found that ion transport and other neuronal processes were reduced in tissue exposed to hypoxia and reoxygenation, which was interpreted as an energy-saving strategy of the seal brain. Further, these authors observed no differential expression of key glycolytic genes that are considered rate limiting, but 3 other glycolytic genes and the monocarboxylate transporter *Mct4* were upregulated, indicating a possibly increased reliance on anaerobic metabolism, in response to hypoxia. Krüger et al. (2020) compared brain transcriptomes of the killer whale (*Orcinus orca*), the long-finned pilot whale (*Globicephala melas*), the bowhead whale (*Balaena mysticetus*) and the minke whale (*Balaenoptera acutorostrata*) to the cattle (*Bos taurus*) and observed a high expression of genes related to aerobic metabolism and detoxification of reactive oxygen species in these cetaceans. Taken together, these studies suggest that diving mammals have evolved adaptations to hypoxia through adjustments of cerebral energy metabolism, but that seals and whales may differ in their regulation of aerobic metabolism. In an immunohistochemistry study using the visual cortex of hooded seals, rats and mice, Mitz et al. (2009) localized cytochrome c, a marker of oxidative metabolism, mainly in fibrillary astrocytes of the seal, while cytochrome c was predominantly found in the neurons of the rodent brain. These results suggest that selection has favored an unusual labor division between neurons and glia cells in the hooded seal brain, making a study of every cell type separately a compelling task.

In this study, we compare the transcriptomes of isolated neurons from the visual cortex of hooded seals with those of mice, as separated using laser-capture microdissection (LCM). We identified gene ontology (GO) terms that our RNA-seq data were enriched for, and studied the more strongly enriched GO terms in greater detail. We focused on anaerobic and aerobic metabolism, and on cost-intensive processes essential to brain function, e.g., neurotransmitter cycling and synaptic signal transmission. We also analyzed the antioxidant defense system that is set to combat reactive oxygen species (ROS). Since there are only whole tissue transcriptomes available for cetaceans, it is difficult to assess whether prominent adaptations recognized in the hooded seal neuronal transcripts are also valid in other marine mammals. We have nevertheless analyzed whale brain transcriptomes, from the long-finned pilot whale (*Globicephala melas*), the killer whale (*Orcinus orca*), the bowhead whale (*Balaena mysticetus*) and the minke whale (*Balaenoptera acutostrata*) that were all used in Krüger et al. (2020), but now with adjusted mapping parameters and also supplemented with data from 2 long-finned pilot whales that were sequenced as part of the present study.

## Materials and Methods

### Animals and Sampling

Hooded seals (*Cystophora cristata*) were captured in March 2016 (*n* = 3 adult females), in the pack ice of the Greenland Sea under permits from relevant Norwegian and Greenland authorities. The animals were euthanized immediately following capture, by sedation with intramuscular injection of zolazepam/tiletamine (1.5–2.0 mg per kg of body mass), followed by catheterization of the extradural intravertebral vein and i.v. injection of an overdose of pentobarbital (Euthasol vet., Le Vet B.V., Netherlands; 30 mg per kg of body mass). All animal handling was in accordance with the Norwegian Animal Welfare Act and with approvals from the National Animal Research Authority of Norway (permits nos. 5399 and 7247). Adult female mice (*Mus musculus*, C57/BL6, *n* = 3) were a gift by Prof. Dr. Christian Lohr (University of Hamburg, Hamburg, Germany) and were anesthetized with 1 ml isoflurane (Forene, Abbott, Germany) in a chamber and decapitated. All animals were handled according to the EU Directive 63 (Directive 2010/63/EU). Fresh tissue of the visual cortex was immediately frozen in liquid nitrogen and transferred to –80°C to be stored for subsequent usage. Hooded seals and mice were adult females and sampling took place within a few days in March for seals and within a day in mice, so neither gender nor season varied between individuals.

### Tissue Section Preparation and Neuronal Staining

The frozen tissue of the hooded seals and mice were cryo-sectioned at 10 μm thickness using a Cryostat CM 1950 (Leica, Wetzlar, Germany). The tissue sections were placed onto 1.0 PEN (D) slides (Carl Zeiss Microscopy, Oberkochen, Germany) that were exposed to UV for 30 min prior usage to remove RNases. Sections were stored at –80°C for a maximum of 2 days. The neurons were identified using a rapid staining with 1% cresyl violet acetat (Roth, Karlsruhe, Germany), which is an alkaline stain that binds acid components such as DNA and RNA. The perikaryon of neuronal cells contains a high number of endoplasmic reticula with rRNA that is stained by cresyl violet (Supplementary Figure 1). Cresyl violet is a commonly used stain to identify neurons (e.g., Coyle and Schwarcz, 1976; Ahmed et al., 2018; Mehrabadi and Sadr, 2020; Clément et al., 2021) and we diluted it in 50% ethanol in diethylpyrocarbonate-treated (DEPC) water, combined with an alcoholic dehydration of the tissue sections using ethanol diluted in DEPC water, to preserve the RNA integrity. Staining solutions were prepared in RNase free conditions and kept on ice. Slides were removed from –80°C (dry ice) and immediately stained without allowing the tissue to thaw. The sections were incubated in (1) 75% ethanol for 30 s, (2) cresyl violet for 30 s, (3) DEPC water for 5 s, (4) 75% ethanol for 30 s, (5) 95% ethanol for 30 s and (6) 100% ethanol for 30 s. For every individual, fresh staining solutions were used. The slides were allowed to dry for 1 min at room temperature and were then used directly for laser capture microdissection (LCM).

### Laser Capture Microdissection

laser capture microdissection was performed using a Zeiss PALM Microbeam 4.2. Microscope System and the 20x objective with the following parameters: speed cut: 30%, speed of laser pressure catapulting (LPC): 50%, cut energy: 39%, LPC energy: 69%, focus: 44 and focus delta: 46. The tissue was visualized on the computer interface, and neuronal cells were manually marked and excised and catapulted into adhesive caps using the RoboLPC function. Neurons were identified by their purple color as a result of the cresyl violet staining. To keep the risk of RNA destruction to a minimum, LCM was performed for only 2.5 min per tissue section. Each slide held four sections, resulting in a total LCM duration of 10 min per slide. Excised cells were collected in AdhesiveCap 500 clear (Carl Zeis Microscopy, Göttingen, Germany) and immediately frozen on dry ice and kept at –80°C until RNA extraction. For each individual, a total of four slides were subjected to LCM and cells were collected in a fresh AdhesiveCap.

### RNA Isolation and Quality Control

Total RNA from the laser-captured neurons was extracted employing the RNeasy Micro Kit (Qiagen, Hilden, Germany) according to the instructions of the manufacturer, with the optional addition of 1% β-mercaptoethanol to the lysis buffer. Additionally, cells were carefully grinded in RLT-buffer with a plastic pistil before vortexing. To yield an RNA concentration as high as possible, the laser-captured cells of all four slides of an individual were pooled per cell type onto one column and eluted in 12 μl of RNase-free water. The RNA concentration and the RIN-score indicating the RNA quality were assessed using the Agilent 4200 TapeStation System and the High Sensitivity RNA ScreenTape Assay (Agilent Technology, Santa Clara, CA, United States).

### Sequencing and Quality Trimming

An ultra-low input RNA-seq library preparation for paired-end sequencing of 150 bp was generated from < 10 pg RNA from neurons of the hooded seal and the mouse. Sequencing was performed on an Illumina HiSeq 4000 platform with an output of ∼70 million reads per sample (Genewiz, Leipzig, Germany). Sequence quality analysis was performed using the CLC Genomics Workbench version 11.0.1 (Qiagen, Venlo, Netherlands). For quality trimming, reads with more than 2 ambiguous bases or a mean Phred score below 35 were removed. Additionally, the first 20 5‘- terminal nucleotides and reads shorter than 30 nucleotides were discarded. Further, the adapters were trimmed (mismatch cost 2, gap costs 3, internal match 15). The raw sequence files are available at the NCBI Sequence Read Archive (SRA) from hooded seal neurons and mouse neurons (PRJNA785765, see Supplementary Table 1 for SRA accession numbers).

### Expression Analysis via RNA-seq

In order to identify transcripts differentially expressed in neurons of hooded seals as compared to mice, the cell-type specific transcriptomes were compared. For that purpose, the quality-trimmed reads were mapped against the NCBI human (*Homo sapiens*) genome (assembly GRCh38.p13) that served as a reference genome. Mice and hooded seals have a similar evolutionary distance to humans^1^, which allows for a comparison of the RNA-seq analyses. The mapping was carried out using the RNA-seq algorithm of the CLC Genomics Workbench v.10.0.1 that uses ELAND for map-placing, as described in Mortazavi et al. (2008). Only reads that matched 75% of the read length and 75% of the nucleotides to the reference genome were included in the mapping. Paired distance ranged from 29 to 455 bp in seal samples and 15 – 572 bp in mouse samples. Gene expression was determined as TPM (Transcripts Per Kilobase Million mapped reads). Only reads that mapped uniquely in the genome were included to calculate TPM values. A Principal Component Analysis (PCA) of the mapped reads in neurons of both species was conducted using the CLC Genomics workbench with default parameters.

In order to verify the ambiguous results of glycolysis, transcripts were additionally mapped to the mouse genome as a reference (GRCm38.97). While for the hooded seal 70% of the read length had to match 70% of the nucleotides to account for the evolutionary distance between the two species, mapping parameters for the mouse transcripts were more stringent and 95% of the read length had to match 95% of the nucleotides.

### Differential Expression Analysis

The differential gene expression analysis was accomplished using the CLC Genomics workbench and the Exact Test (Robinson and Smyth, 2007) implemented in the empirical analysis tool. The unique gene reads were normalized to the total reads of each sample and used as count data. A total count cut-off of five reads was applied and p-values were corrected for multiple testing using the False Discovery Rate (FDR).

### Gene Ontology Analysis

Gene Ontology (GO) analyses were performed using the PANTHER Overrepresentation Test (PANTHER version 16.0 Released 2020-12-01^2^) and human as the reference list. For the GO analysis, differentially expressed genes with p_FDR_ ≤ 0.05, a TPM ≥ 5 and a fold change (FC) ≥ 2 or ≤ –2 were considered. Overrepresentation was determined in the GO-slim category “Biological process” using Fisher’s Exact Test and the FDR as correction for multiple testing.

### Analysis of Selected Gene Ontology Terms and Related Pathways

Based on the GO analysis, selected GO terms and related pathways, e.g., glutamine-glutamate cycle, were analyzed in more detail. To best represent all genes in these GO terms or pathways, a less stringent selection of genes with p_FDR_ ≤ 0.05, TPM ≥ 1 and a FC ≥ 2 or ≤ –2 were considered. Genes were extracted from the dataset using the statistics program R version 3.5.1 (R Core Team, 2013) and the packages knitr (Xie, 2013) and Dplyr (Wickham and Francois, 2014).

### Sampling, Sequencing and Expression Analysis in Whales

Raw reads of the transcriptomes of brain samples of four whale species and cattle (*Bos taurus*) were retrieved from NCBI (PRJNA506903, see Supplementary Table 2 for SRA accession numbers). Additionally, the visual cortex and cerebellum of 2 long-finned pilot whales (*Globicephala melas*) were sampled opportunistically according to the procedures described in Krüger et al. (2020) and with permission of the German law (*Tierisches Nebenprodukte Beseitigungsgesetz*). Regulations of the Convention on Biological Diversity (CBD) and Convention on International Trade in Endangered Species of Wild Fauna and Flora (CITES) were followed, and the appropriate permits were obtained (Permit number: E-01456/17). RNA extraction and sequencing of the two sampled long-finned pilot whales were performed as described in Krüger et al. (2020). Briefly, total RNA was extracted using the peqGOLD Trifast (PEQLAB, Erlangen, Germany) and Crystal RNA Mini Kit (Biolab Products, Bebensee, Germany). After assessing RNA integrity and quantity with the Agilant TapeStation System (Agilent Technology, Santa Clara, CA, United States), paired-end sequencing (PE 150) was performed on a NextSeq 500 platform (StarSEQ, Mainz, Germany) with an output of ∼60 million reads per sample. Raw reads of the newly sequenced long-finned pilot whales are available at the NCBI Sequence Read Archive (SRA) (PRJNA785431, Supplementary Table 2). In total, our data consisted of transcripts from the visual cortex (*n* = 3) and cerebellum (*n* = 3) of long-finned pilot whales, the visual cortex of the killer whale (*Orcinus orca*, *n* = 1), an unspecified brain region of a minke whale (*Balaenoptera acutostrata*, *n* = 1), the cerebellum of a bowhead whale (*Balaena mysticetus, n* = 1) as well as the visual cortex (*n* = 3) and the frontal lobe (*n* = 2) of cattle.

Sequence analysis of the already available raw reads and the newly sequenced reads was performed using the CLC Genomics Workbench version 11.0.1 (Qiagen, Venlo, Netherlands). For quality trimming, reads with more than 2 ambiguous bases and a Phred score below 15 were removed. Additionally, the first 15 5′-terminal nucleotides and reads shorter than 30 nucleotides were discarded.

Trimmed reads of whales and cattle were mapped against the human genome (GRCh38.p13) retrieved from NCBI. Only reads that matched 75% of the read length and 75% of nucleotides of the reference were included in the mapping. Reads with non-specific matches were ignored and the paired-read distance was calculated automatically. Gene expression was determined as RPKM (Reads per kilo base per million mapped reads). The differential gene expression analysis and the GO analysis were performed as for the hooded seal data. Differentially expressed genes with p_FDR_ ≤ 0.05, a RPKM ≥ 5 and FC ≥ 2 or ≤ -2 were considered for GO analysis. For more detailed analyses of specific pathways, genes with RPKM ≥ 1 were considered.

Additionally, we performed a differential expression analysis between hooded seal neurons and cattle (neurons and glia). Analyses were performed as described for the other comparisons with genes having a TPM value ≥ 5, FC ≥ 2 or ≤ –2 for the GO analysis and genes having a TPM value ≥ 5, FC ≥ 2 or ≤ –2 for the analysis of specific pathways.

### Positive Selection

To test whether differentially expressed genes may additionally have evolved functional changes in diving mammals, we tested the 20 genes with the top 10 and bottom 10 fold changes in hooded seal neurons compared to mice (Tables 1, 2) for positive selection. To explore convergent evolution, we tested the same genes for positive selection in cetaceans. We compared the hooded seal, the Weddell seal (*Leptonychotes weddelli*), the southern elephant seal (*Mirounga leonina*) and the walrus (*Odobenus rosmarus*) to the dog (*Canis lupus familiaris*), the ferret (*Mustela putorius furo*), the giant panda (*Ailuropoda melanoleuca*) and the polar bear (*Ursus maritimus*). Among cetaceans, we selected the sperm whale (*Physeter catodon*), the minke whale (*Balaenoptera acutorostrata*), the long-finned pilot whale (*Globicaphala melas*), the bottlenose dolphin (*Tursiops truncatus*) and the Chinese river dolphin (*Lipotes vexillifer*) and compared them to cattle (*Bos taurus*), sheep (*Ovis aries*), the wild boar (*Sus scrofa*), and the red deer (*Cervus elaphus*). Sequences were retrieved from NCBI with exception of the hooded seal, for which sequences were extracted from the data of this study. Alignments were created in TranslatorX (Abascal et al., 2010) that additionally creates a MAFFT amino acid alignment to improve alignment quality. Selection pressures were inferred using the branch-wide unrestricted statistical test for episodic diversification (BUSTED) (Murrell et al., 2015) and the adaptive branch-site random effects likelihood (aBSREL) model (Pond and Frost, 2005) on the Datamonkey server^3^. In both methods, pinnipeds and cetaceans were denoted as foreground branches, in which some sites might be positively selected, whereas in the terrestrial background branches, selection was assumed to be absent. While BUSTED estimates whether a gene has undergone positive selection in at least one site in at least one branch, aBSREL determines for each foreground branch whether a proportion of sites have undergone positive selection. Only if both methods detected selection pressure, we considered a gene positively selected.

**TABLE 1:** Top 10 differentially expressed genes: genes with the highest fold change in the neurons of the hooded seal visual cortex compared to the mouse.

Gene symbol	Fold change	FDR	Mouse TPM	Seal TPM	Gene name	Function
*S100B*	82.11	1⋅10^–136^	5.88	479.40	S100 Calcium Binding Protein B	Ca^2+^-dependent regulation of many physiological processes
*MAGEE2*	31.71	8.1⋅10^–121^	7.87	248.11	MAGE Family Member E2	Unknown
*MT-ATP6*	29.11	1.1⋅10^–58^	6.39	185.28	Mitochondrially Encoded ATP Synthase Membrane Subunit 6 (Complex V)	Electron transport chain, Complex V
*BLVRB*	19.77	3.9⋅10^–55^	6.20	122.32	Biliverdin Reductase B	broad specificity oxidoreductase, heme metabolism
*MT-ND1*	19.21	5.2⋅10^–67^	21.03	399.34	MT NADH:Ubiquinone Oxidoreductase Core	Electron transport chain, Complex I
*UQCR10*	18.96	2.9⋅10^–65^	8.36	157.71	Ubiquinol-Cytochrome C Reductase, Complex III Subunit X	Electron transport chain, Complex III
*ENC1*	18.82	2.7⋅10^–123^	14.47	270.85	Ectodermal-Neural Cortex 1	Neuronal process formation, oxidative stress
*NDUFA2*	17.79	4.4⋅10^–48^	9.49	167.97	NADH:Ubiquinone Oxidoreductase Subunit A2	Electron transport chain, Complex I
*BLVRA*	17.53	1.9⋅10^–68^	8.88	155.23	Biliverdin Reductase A	Heme metabolism
*CHGA*	17.08	1.3⋅10^–93^	11.39	181.10	Chromogranin A	Neuroendocrine secretion

**TABLE 2:** Top 10 differentially expressed genes: genes with the most negative fold change in the neurons of the hooded seal visual cortex compared to the mouse.

Gene symbol	Fold change	FDR	Mouse TPM	Seal TPM	Gene name	Function
*RRP1*	–12.48	5.2⋅10^–102^	70.43	5.62	Ribosomal RNA Processing 1	Generation of 28S rRNA
*GAA*	–13.45	1.7⋅10^–82^	85.99	6.36	Alpha Glucosidase	Glucose metabolism
*CTXN1*	–14.50	2.5⋅10^–60^	103.44	7.16	Cortexin 1	Neuronal signaling during brain development
*SYP*	–14.94	0	398.33	26.55	Synaptophysin	Component of synaptic vesicles
*PITPNA*	–15.19	2.4⋅10^–127^	186.03	12.13	Phosphatidylinositol Transfer Protein Alpha	Phospholipase C signaling
*NAPA*	–16.32	7.8⋅10^–63^	106.20	6.47	NSF Attachment Protein Alpha	Intracellular vesicle transport
*NSF*	–16.89	0	364.98	21.17	*N*-Ethylmaleimide Sensitive Factor, Vesicle Fusing ATPase	Intracellular vesicle transport
*CAMTA2*	–17.48	2⋅10^–63^	94.34	5.36	Calmodulin Binding Transcription Activator 2	Possibly involved in cardiac growth and tumor suppression
*CACNG3*	–19.27	1.1⋅10^–108^	220.53	11.42	Calcium Voltage-Gated Channel Auxiliary Subunit Gamma 3	Regulation of AMPA-selective glutamate-receptors
*CPLX1*	–19.96	6.3⋅10^–113^	133.66	6.37	Complexin 1	Positive regulation of exocytosis

*Negative fold changes indicate a lower expression in seal neurons than in the mouse.*

## Results

### Separation and RNA-Extraction of Hooded Seal Neurons

Neurons were stained with cresyl-violet and separated using LCM (Supplementary Figure 1). RNA quantity and quality were assessed with the Agilent 4200 TapeStation System. Neurons yielded ∼348 pg/μl of RNA and a mean RIN value of 7.3.

### Transcriptomes of Neurons

We generated ∼70 million paired-end Illumina reads per sample of 150 nt length. Raw read quality was good (Phred score ∼37) and reads with a Phred score below 35 were removed during the trimming process. The mapping using the human genome as a reference resulted in ∼32 million mapped reads in mouse and ∼26 million reads in hooded seal samples (Supplementary Table 2).

**FIGURE 1:**
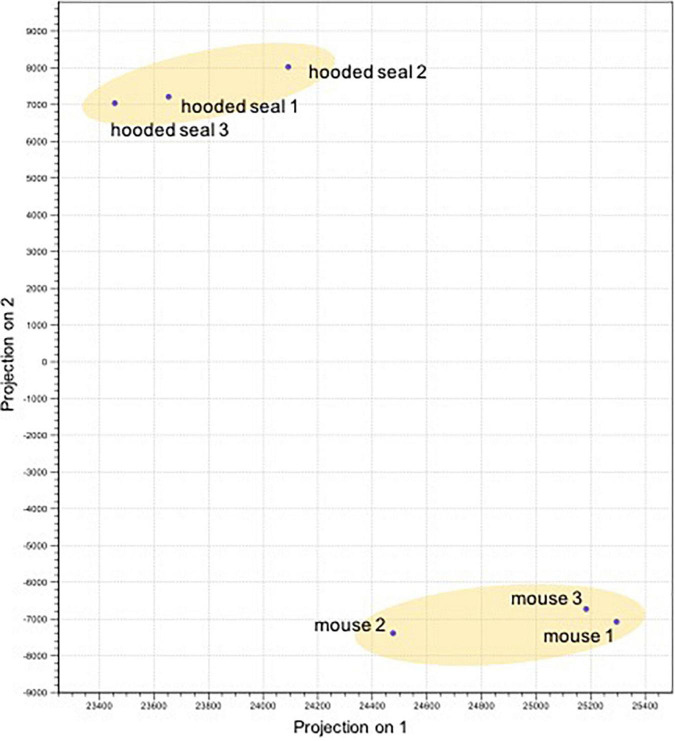
Principal component analysis of the differentially expressed genes (normalized expression values) of the neurons in the visual cortex of hooded seals (*Cystophora cristata*, *n* = 3) and mice (*Mus musculus*, *n* = 3).

The PCA illustrated that neurons of both species grouped separately and thus, showed distinct expression patterns (Figure 1). The differential expression analysis resulted in 7310 differentially expressed genes (DEGs) in neurons. For GO analyses, only DEGs with a TPM ≥ 5 and a fold change (FC) ≥ 2 or ≤ –2 were considered.

### Genes More Highly Expressed in Hooded Seal Neurons Were Enriched for Mitochondrial Organization and Function

**FIGURE 2:**
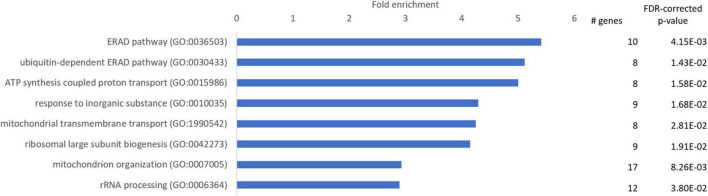
Enriched GO-slim terms in the category “biological processes” in the neurons of the visual cortex of hooded seals compared to mice. Genes used for analysis were significantly more highly expressed in hooded seals (fold change ≥ 2). The number of hooded seal genes in every GO-slim term and the FDR-corrected *p*-value are presented.

To identify GO terms overrepresented in our data, we performed a Panther overrepresentation analysis in the category “GO slim biological processes” and we present the top 10 main GO terms that genes were significantly enriched for in hooded seals compared to mice. Most of these GO-terms were related to mitochondrial organization and function (ATP synthesis coupled proton transport, mitochondrial transmembrane transport, mitochondrion organization), followed by endoplasmatic reticulum (ER)-associated protein degradation (ERAD) pathway, ubiquitine-dependent ERAD pathway and ribosome-associated processes (ribosomal large subunit biogenesis, rRNA processing) (Figure 2).

We further examined the 10 most highly expressed genes in seal compared to mouse neurons (Table 1). The gene with the highest fold change (FC) was S100B, a calcium binding protein that regulates numerous physiological processes. Four of the genes code for components of the electron transport chain (*MT-ATP6, MT-ND1, UQCR10, NDUFA2*), two genes are involved in heme metabolism (*BLTVA, BLRVB*), one gene plays a role in neuroendocrine secretion (*CHGA*) and *ENC1* is involved in neuronal process formation and oxidative stress.

### Genes With Lower Expression in Seal Neurons Were Enriched for Neurotransmission and Protein Metabolism

**FIGURE 3:**
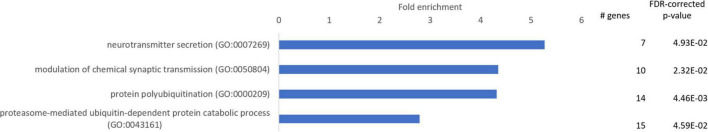
Enriched GO-slim terms in the category “biological processes” in the neurons of the visual cortex of hooded seals compared to mice. Genes used for analysis were significantly less expressed in hooded seals (fold change ≤ –2). The number of hooded seal genes in every GO-slim term and the FDR-corrected *p*-value are presented.

Genes with a reduced expression in seal compared to mouse neurons (FC ≤ –2) were enriched for 4 GO-slim terms related to neurotransmission (neurotransmitter secretion, modulation of chemical synaptic transmission), protein degradation (protein polyubiquitination) and protein catabolism (proteasome-mediated ubiquitin-dependent protein catabolic process) (Figure 3).

Among the 10 genes with the lowest FC, we found genes that concern intracellular vesicles (*SYP*), are involved in intracellular vesicle transport (*NAPA, NSF*), signaling (*CTXN1, PITPNA, CACNG3*), glycogen degradation (*GAA*) and generation of 28s rRNA (*RRP1*). The gene with the lowest FC of –20 is a positive regulator of exocytosis (*CPLX1*) (Table 2). Although *GAA* is among the genes with the lowest FC, the majority of genes involved in glycogenolysis (GO term “glycogen catabolic process”) were, in fact, more highly expressed in hooded seal neurons than in mice (5 of 8 genes).

### Reduced Synaptic Transmission in the Hooded Seal

The GO analysis and the analysis of the top 10 differentially expressed genes point to a reduced signal transmission and an elevated activity of the electron transport chain in seal neurons. We investigated these processes in more detail by assessing the expression of genes belonging to functionally related GO terms and pathways. To assess these GO terms as comprehensively as possible, we considered genes with TPM values ≥ 1, FC ≥ 2 or ≤ –2 and p_FDR_ ≤ 0.05.

**FIGURE 4:**
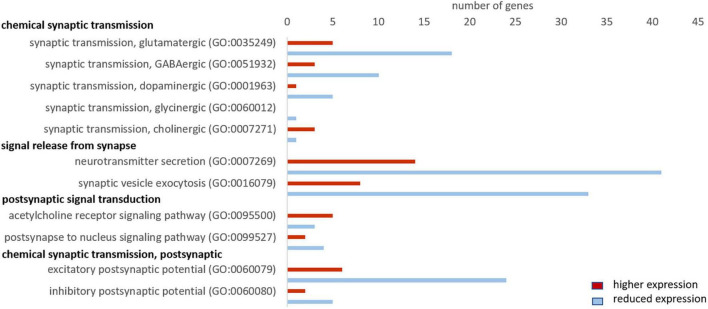
Genes related to neuronal signaling that show a higher (red) and lower expression (blue) in hooded seals compared to neurons of mice. Only genes with TPM values ≥ 1 and FC ≥ 2 or ≤ –2 were considered.

First, we evaluated GO terms related to chemical synaptic transmission. The majority of genes showed a lower expression in seal than in mouse neurons. This concerned 78% (18 of 23 genes) related to “synaptic transmission, glutamatergic,” 80% of genes related to “synaptic transmission, GABAergic” (10 of 13 genes) and 5 out of 6 genes related to “synaptic transmission, dopaminergic” (Figure 4). Of the term “synaptic transmission, glycinergic” we found only one gene in our dataset that was less expressed in seal neurons and “synaptic transmission, cholinergic” yielded four genes, of which only 1 was less expressed in seals. We then assessed the GO terms related to signal release from synapse and found gene expression to be reduced in 75% of genes (41 of 55) in “neurotransmitter secretion,” while 80% of the genes (33 of 41) in “synaptic vesicle exocytosis” were less expressed in seal neurons. In the term “postsynapse to nucleus signaling pathway,” related to postsynaptic signal transmission, 4 of 6 genes were less expressed in seals, while in “acetylcholine receptor signaling pathway” only 3 of 5 genes had lower TPM values in seal than in mouse neurons. Finally, 80% (24 of 30) and 71% (5 of 7) of genes in the terms “excitatory postsynaptic potential” and “inhibitory postsynaptic potential,” respectively, were less expressed in seals than in mice (Figure 4).

### Reduced Glutamate Release and Increased Glutamate Uptake in the Hooded Seal

**FIGURE 5:**
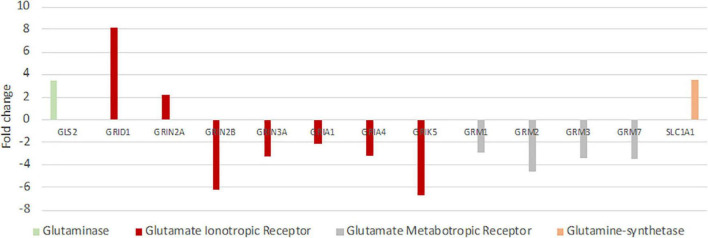
Differential expression of genes playing key roles in glutamatergic synaptic transmission and the glutamine-glutamate-cycle in hooded seals compared to neurons of mice. Positive fold changes represent a higher expression in seal neurons.

Since failure of glutamate reuptake from the synaptic gap during hypoxia causes neurotoxicity as part of the excitotoxic cascade (e.g., Swanson et al., 1995; Lutz et al., 2003), adaptations in the glutamate-glutamine cycle could be one major molecular mechanism to cope with hypoxic conditions. Thus, we investigated this cycle in greater detail. Genes involved in the positive regulation of “synaptic transmission, glutamatergic” were typically less expressed in seal neurons (up to FC –20), whereas genes playing a role in the negative regulation were more highly expressed in seals (up to FC 3.6) (Supplementary Table 3). Further, almost all glutamate receptors show a reduced expression in seal neurons (Figure 5). Additionally to the receptors presented in Figure 5, three more glutamate receptors were significantly less expressed in seals neurons but not quite meeting our criteria of FC ≤ –2 (*GRIN1*: FC = –1.5, *GRIA2*: FC = –1.9, *GRIA3*: FC = –1.9). Glutaminase (*GLS*, FC = –1.4), hydrolyzing glutamine to glutamate in neurons, was significantly less expressed in seal neurons. The neuronal glutamate transporter, crucial in removing glutamate from the synaptic cleft, was more highly expressed in seal neurons (*SLC1A1*, FC = 3.5) (Figure 5).

### Enhanced Aerobic Metabolism in Hooded Seal Neurons

**FIGURE 6:**
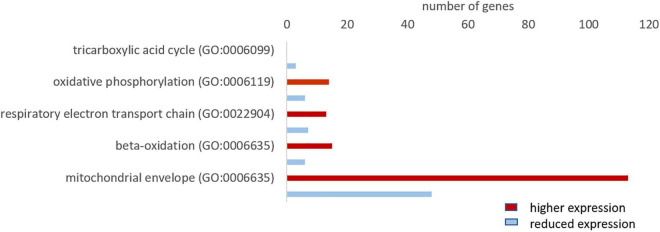
Total number of genes related to aerobic metabolism with a higher (red) and lower expression (blue) in the hooded seal cortical neurons compared to mouse neurons.

Since oxygen is vital for the production of ATP in the mitochondria, we investigated whether the hypoxia-tolerant seal neurons show adaptations in their aerobic metabolism. The GO analysis revealed that genes related to mitochondria organization and function were more highly expressed in the hooded seal (Figure 2). To get a more detailed picture, we analyzed related GO terms and found that ∼70% of the genes related to “oxidative phosphorylation,” “respiratory electron transport chain” and “beta-oxidation” were more highly expressed in seal neurons than in mouse neurons (Figure 6). For “tricarboxylic acid cycle” we found only 3 genes that matched our criteria (Figure 6), which might not be very informative. When setting a lower FC cutoff (FC ≥ 1.5 or ≤ –1.5), we found almost even numbers, i.e., 6 and 5 genes being more highly and less expressed, respectively, in seal neurons. Moreover, 70% of genes related to the GO term “mitochondrial envelope” were more highly expressed in seal than in mouse neurons (Figure 6), suggesting that not only mitochondrial function, but also mitochondrial numbers, are enhanced in seal neurons.

### Elevated Antioxidant Expression in Hooded Seal Neurons

**FIGURE 7:**
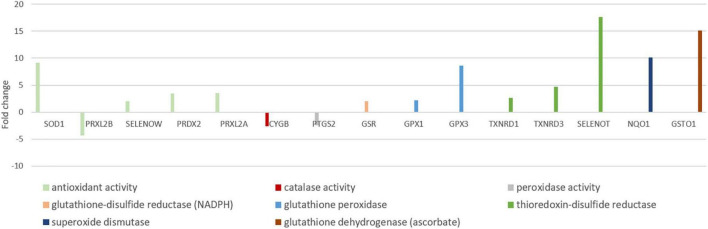
Antioxidant genes differentially expressed in neurons of mouse and hooded seal visual cortex. Positive fold changes represent a higher expression in hooded seal neurons.

Antioxidants are crucial in the defense against reactive oxygen species (ROS), which are mainly produced during reoxygenation (Lutz et al., 2003; Chen et al., 2018), after the seal surfaces. The majority of the genes in “antioxidant activity (GO:0016209)” were more highly expressed in seal neurons than in mouse neurons (12 in 15 genes). The most highly expressed genes were selenoprotein T (*SELENOT*, FC = 17.6), glutathione-*s*-transferase omega 1 (*GSTO1*, FC = 15.1), superoxide dismutase 1 (*SOD1*, FC = 9.1) and glutathione peroxidase 3 (*GPX3*, FC = 8.5) (Figure 7).

### Possibly Decreased Glycolysis in Hooded Seal Neurons

At first sight, there were no striking differences between mouse and hooded seal neurons regarding the “glycolytic process (GO:0006096)” since the number of genes more highly (10 genes) and less expressed (13 genes) was almost even. However, several genes had stronger negative- than positive-fold changes. When extracting only genes of the glycolysis (Table 3), some enzymes playing key roles for glycolytic rate (Berg et al., 2012) had reduced expression values in seal neurons. We noted that some genes did not meet our usual criterion of FC ≥ 2 or ≤ –2. To verify these results, transcripts were additionally mapped to the mouse as a reference, with similar results (Supplementary Table 4). Further, a neuronal glucose transporter (SLC1A3 or GLUT3), was less expressed in seal neurons (FC = –1.7, p_FDR_ = 0.0003). Lactate dehydrogenase A and B (LDHA, LDHB), which convert pyruvate to lactate, were equally expressed and the monocarboxylate transporter 4 (SLC16A3 or MCT4), which transports lactate out of the cell, was more highly expressed in seal than in mouse neurons (FC = 3, p_FDR_ = 7.10^–6^). We found no differences in “gluconeogenesis (GO:0006094)” between species, with the exception of forkhead box O1 (*FOXO1)*, a transcription factor that upregulates gluconeogenesis, which was down-regulated in seal neurons (FC = –1.7, p_FDR_ = 0.003).

**TABLE 3:** Expression of genes involved in glycolysis in the mouse and hooded seal cortical neurons.

Gene symbol	Gene name	Fold change	FDR	Mouse TPM	Seal TPM
***Part I: glucose to fructose-1,6-bisphosphate***					
**HK1**	Hexokinase 1	–1.5	<0.001	111	75
**PFKL**	Phosphofructokinase, liver	–9.2	0	48	5
**PFKP**	Phosphofructokinase, platelet	1.8	0.001	46	84
ALDOA	Aldolase A	–1.7	4.52e-05	14	8
TPI1	Triosephosphate isomerase 1	1.6	0.022	384	616
***Part II: glyceraldehyde-3-phosphate to pyruvate***					
PGK1	Phosphoglycerate kinase 1	2.0	5.08e-05	147	288
PGAM1	Phosphoglycerate mutase 1	2.5	1.05e-08	82	203
PGAM2	Phosphoglycerate mutase 2	4.2	1.57e-07	3	15
ENO2	Enolase 2	–69	1.15e-181	342	5

*Negative fold changes indicate a lower expression in seal neurons compared to the mouse. Rate-limiting enzymes are in bold letters.*

### Expression Analysis in Whales

#### Reduced Synaptic Transmission in Whale Brains

**FIGURE 8:**
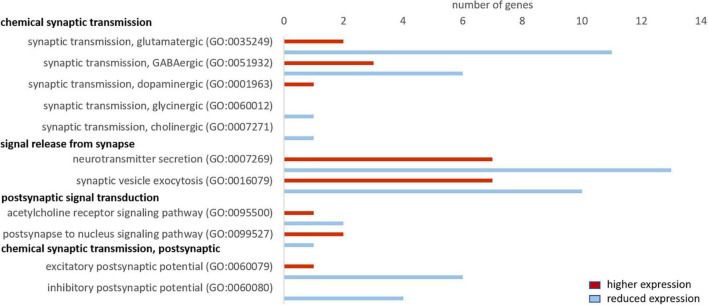
Genes related to neuronal signaling that show a higher (red) and lower expression (blue) in whales compared to cattle. Only genes differentially expressed (fold change ≥ 2, range ≥ 5, RPKM ≥ 1 and FDR-corrected *p*-value ≤ 0.05) were considered.

Cattle and whale brain samples (i.e., both neurons and glia cells represented) yielded ∼50 and ∼47 million trimmed reads, respectively, of which 57% and ∼52%, respectively, mapped to the human genome (Supplementary Table 1). We found 5081 differentially expressed genes with p_FDR_ ≤ 0.05, of which 589 genes had an FC ≥ 2 or ≤ –2 and RPKM ≥ 5 (used for Panther overrepresentation test) and 1562 genes had a FC ≥ 2 or ≤ –2 and RPKM ≥ 1 (used for analysis of related GO terms and pathways). We checked whether our main findings of a reduced synaptic transmission in seal neurons was also evident in cetaceans, which would imply that this is a common characteristic shared by marine mammals. We performed a GO analysis and then analyzed the same GO terms as for the hooded seal data. We found that the majority of genes in GO terms related to chemical synaptic transmission, signal release from synapse, postsynaptic signal transmission and postsynaptic chemical transmission, were less expressed in whale compared to cattle brains (Figure 8). Considering the glutamate signaling in more detail, we found that genes of the GO term “synaptic transmission, glutamatergic” were less expressed in whales. Only four genes of the glutamate-glutamine cycle were present in the whale data. The glutamate receptors *GRIA3, GRM5* and *GRIK1* were less expressed in whales (FC_GRIA3_ = –3.4, pFDR_GRIA3_ < 0.001, FC_GRM5_ = –2.8, pFDR_GRM5_ < 0.001, FC_GRIK1_ = –2.5, pFDR_GRIK1_ < 0.001) and the glutamate transporter *SLC1A6* was more highly expressed in whales (FC = 4.2, pFDR_GRM_ = 0.04) compared to cattle.

#### Enhanced Aerobic Metabolism in Whales

**FIGURE 9:**
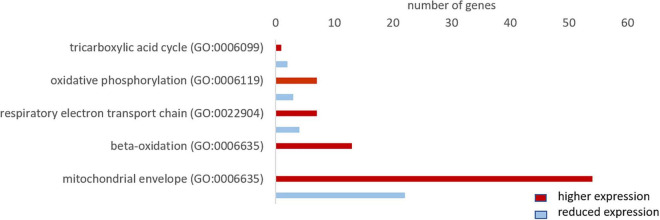
Total number of genes related to aerobic metabolism with a higher (red) and lower expression (blue) in whale brains compared to the cattle. Only genes differentially expressed (fold change ≥ 2, range ≥ 5, RPKM ≥ 1 and FDR-corrected *p*-value ≤ 0.05) were considered.

For the GO term “oxidative phosphorylation,” we found 10 genes, of which 7 were more highly expressed in whales. For “respiratory electron transport chain” the majority of genes (7 in 11), and for “beta oxidation” all of the 13 found genes, were more highly expressed in whales. We only found three genes of the “tricarboxylic acid cycle,” of which 1 was more highly expressed in whales. Further, the majority of genes (54 in 77 genes) of the GO term “mitochondrial envelope” were more highly expressed in whales (Figure 9).

#### Increased Glycolytic Capacity in Whale Brains Compared to Cattle

The GO term “glycolytic process” was represented by 13 genes, of which four genes were less and nine genes were more highly expressed in whales than in cattle. Four of the glycolytic genes presented for the hooded seal data (Table 3) were also present in the whale data. Two of the rate-limiting enzymes, that is *PFKL* and *PFKP*, were more highly expressed in whales (FC_PFKL_ = 3.9, pFDR_GRIA3_ = 0.04, FC_PFKP_ = 4.2, pFDR_GRIA3_ = 0.003). Similarly, *ALDOA* and *PGAM1* were more highly expressed in whales compared to cattle (FC_ALDOA_ = 36.4, pFDR_ALDOA_ < 0.001, FC_PGAM1_ = 3.6, pFDR_ALDOA_ = 0.014).

### Differential Expression Analysis in Hooded Seals Compared to Cattle

Since hooded seals are more closely related to cattle (78 million years) than to mice (96 million years), we additionally performed a differential expression analysis of hooded seal neurons and cattle brain (neurons and glia). The results are very similar to the analysis of hooded seals vs. mice and are presented in the Supplementary material (Supplementary Figures 2–7).

### Positive Selection

We tested whether differential expression is accompanied by a potential functional shift in pinnipeds and cetaceans compared to their terrestrial relatives (Table 4). Among the 10 genes with the highest fold changes in hooded seal neurons compared to mice neurons (Table 1), BUSTED determined that at least one site in the biliverdin reductase B (BLRVB, *p* ≤ 0.001) and Chromogranin A (*CHGA*, *p* = 0.012) has been subjected to positive selection in at least one pinniped branch. aBSREL determined the branches to be the Weddell seal for BLRVB (*p* ≤ 0.001) and the hooded seal for *CHGA* (*p* ≤ 0.001). Among the 10 genes with the lowest expression in hooded seal neurons compared to mice neurons (Table 2), BUSTED found the calcium voltage-gated channel auxiliary subunit gamma 3 (*CACNG3*, *p* = 0.012) and the calmodulin-binding transcription activator 2 (*CAMTA2*, *p* = 0.04) to be positively selected in pinnipeds and aBSREL estimated that positive selection occurred in Weddell seals for *CACNG3* (*p* ≤ 0.001) and in hooded seals and southern elephant seals for *CAMTA2* (*p* ≤ 0.001 for each branch). In cetaceans, none of the above genes experienced positive selection. Among genes with the lowest fold changes in hooded seal neurons compared to mice neurons, BUSTED found the NSF attachment protein alpha (*NAPA*, *p* = 0.04) and the *N*-ehtylmaleimide sensitive factor, vesicle fusing ATPase (*NSF*, *p* ≤ 0.001) to be positively selected in cetaceans and aBSREL determined that positive selection occurred in minke whales for *NAPA* (*p* = 0.0037) and in the Chinese river dolphin for *NSF* (*p* ≤ 0.001).

**TABLE 4:** Positive selection among pinniped and cetacean lineages calculated used BUSTED and aBSREL.

	Pinnipeds				Cetaceans			
Gene symbol	BUSTED		aBSREL		BUSTED		aBSREL	
	*p*-value	Alignment (%)	Branch	*p*-value	*p*-value	Alignment (%)	Branch	*p*-value
BLRVA	–	–	–	–	–	–	–	–
BLRVB	<0.001	15.06	Weddell seal	<0.001	–	–	–	–
CACNG3	0.026	1.61	Hooded seal	<0.001	–	–	–	–
CAMTA2	0.04	2.79	Hooded seal, s. elephant seal	<0.001	–	–	–	–
CHGA	0.012	66.65	Hooded seal	<0.001	–	–	–	–
CPLX1	–	–	–	–	–	–	–	–
CTXN1	–	–	–	–	–	–	–	–
ENC1	–	–	–	–	–	–	–	–
GAA	–	–	–	–	–	–	–	–
MAGEE2	–	–	–	–	–	–	Minke whale	0.0271
MT–ATP6	–	–	Hooded seal	<0.001	–	–	–	–
MT–ND1	NA	NA	NA	NA	NA	NA	NA	NA
NAPA	–	–	–	–	0.004	0.09	Minke whale	0.0037
NDUFA2	–	–	–	–	–	–	–	–
NSF	–	–	–	–	<0.001	2.03	Chinese river dolphin	<0.001
PITPNA	–	–	Hooded seal	0.0128	–	–	–	–
RRP1			–	–	–	–	–	–
SYP	–	–	–	–	–	–	–	–
UBQR10	–	–	–	–	NA	NA	NA	NA

*Alignment (%) of the BUSTED approach is the proportion of the alignment for which diversifying positive selection (ω > 1) was detected. P-values are corrected for multiple testing. NA denotes the genes for which sequences were not available at NCBI and (–) indicates that no positive selection was identified. S100B was found to be strongly conserved in pinnipeds and cetaceans using BUSTED (Geßner et al., 2020) and thus, was omitted in this study. For gene names, please see Tables 1, 2.*

## Discussion

Hooded seal neurons show a remarkable intrinsic hypoxia tolerance (Folkow et al., 2008; Geiseler et al., 2016). When exploring the molecular mechanisms of the neuronal hypoxia tolerance, gene expression profiles of specific cell types are more informative than whole-tissue transcriptomes. We separated hooded seal and mouse cortical neurons using LCM to yield neuron-specific expression profiles from both species. To test whether the specific expression pattern of hooded seal neurons might be a characteristic shared by all marine mammals, we compared seal neuron data with whole-tissue (neurons and glia cells) transcriptomes of four whale species.

### Reduced Synaptic Transmission in Hooded Seal Neurons

**FIGURE 10:**
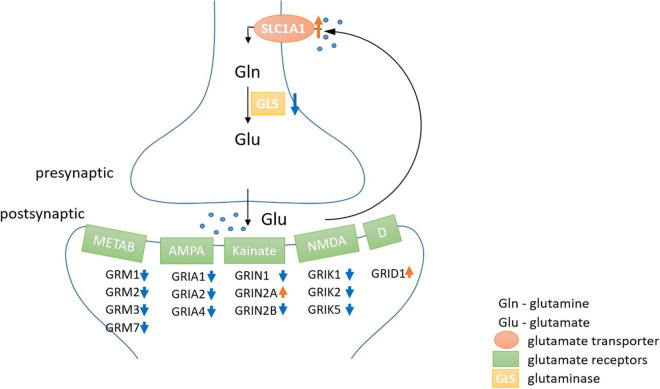
The neuronal part of the glutamate-glutamine-cycle. The majority of metabotropic (METAB) and ionotropic [AMPA, Kainate, NMDA and Delta type (D)] glutamate receptors were lower expressed (blue arrow) in hooded seal neurons, while higher expression is indicated by a red arrow. Astrocytes play an important role in the glutamate-glutamine-cycle, but were not analyzed in this study and thus, were omitted in the scheme.

At rest, the brain consumes 20% of the oxygen and 25% of the glucose in the human body (Kety, 1957; Sokoloff, 1960; Rolfe and Brown, 1997). This high energy expenditure is explained by cerebral functions that include the maintenance and restoration of ion gradients after action and synaptic potentials, the reuptake of neurotransmitters from the synaptic cleft and the recycling of neurotransmitters (Attwell and Laughlin, 2001). Recordings in rat (*Rattus norvegicus*) hippocampal neurons revealed that synaptic potentials, rather than action potentials, account for most of the cellular energy requirement (Alle et al., 2009). We found that genes related to neurotransmission were significantly less expressed in the hooded seal. This included genes related to the modulation of chemical synaptic transmission, neurotransmitter secretion, signaling, vesicle structure and vesicle transport and the regulation of exocytosis. Synaptic transmission genes were also less expressed in four whale species. Overall, our data indicate a reduced intensity of processes related to signal transmission in marine mammals. Glutamatergic synapses represent ∼80% of the excitatory synapses in the cortical gray matter of mammals (presumably including seals and whales) and consequently account for most of the energy used (Bélanger et al., 2011). Further, glutamate overexpression (excitotoxicity) plays a central role in ischemic brain damage. Inhibition or suppression of synaptic transmission, e.g., via blocking of postsynaptic glutamate receptors, decreases neurosensitivity to hypoxia and ischemia in rat neuronal cultures (Rothman, 1983; Nowak et al., 1984) and plays an important protective role in some vertebrate species that display particularly high tolerance toward hypoxia/anoxia (e.g., Nilsson et al., 1991; Lutz et al., 2003). Failure to remove glutamate from the synaptic cleft due to ATP deficiency during hypoxia is a major contributor to neurotoxicity (e.g., Swanson et al., 1995). For this reason, we focused on genes involved in glutamatergic transmission and found the vast majority of them to be less expressed in hooded seals compared to mice. Although not many genes of the glutamate-glutamine cycle were present in whales, or matched our criteria of cutoff, the lower expression of glutamate receptors and higher expression of glutamate transporter in cetaceans were in concordance with our findings in the hooded seal. These results may indicate a reduction of glutamatergic signaling in the cortex of marine mammals (Figure 10), which would likely decrease energy requirements and limit the risk of neuro-/excitotoxicity during hypoxia exposure. Another adaptation that possibly limits the risk of neuro-/excitotoxicity in hypoxia, is the 82-fold higher expression of the S100B gene (Table 1). The S100B protein is known to bind Ca^2+^ and it has been suggested that its high expression in marine mammals may help prevent excitotoxicity by reducing free intracellular Ca^2+^ and thereby attenuate continued release of glutamate and other neurotransmitters (Geiseler et al., 2016).

*GRIN2B*, an ionotropic glutamate receptor, was substantially less expressed (FC = –6.2) in hooded seal than in mouse neurons. In mouse pups, protracted gestational hypoxia triggered downregulation of several subunits of glutamate receptors, which appeared to prevent white matter damages. In rat pups, the same hypoxic conditions did not reduce glutamate receptor expression, and white matter damages were observed (Fontaine et al., 2008). Consequently, while a downregulation of receptor expression might be advantageous when facing hypoxia, the ability to alter gene expression in response to hypoxia seems to be species-dependent. Thus, differences in the expression of *GRIN2B* in mice and rats appeared to strongly affect their susceptibility to hypoxia-induced white matter damage (Fontaine et al., 2008) and lack of *GRIN2B* may therefore also be important for the hypoxia-tolerance displayed by hooded seal neurons. Glutamate transporters, which are responsible for the reuptake of glutamate from the synaptic cleft, are mainly present in astrocytes (Levy et al., 1998). However, a higher expression, and thus, likely a higher density, of the neuronal glutamate transporter *SLC1A1*, in neurons may contribute to efficient removal of glutamate from the synaptic cleft.

In summary, decreased densities of glutamate receptors and enhanced Ca^2+^ -binding by S100B, may both limit the magnitude of glutamatergic excitation, should the removal of glutamate from the synaptic cleft fail due to insufficient ATP-production during hypoxia. We note that two of 11 glutamate receptors, *GRID1* (TPM_seal_ = 21) and *GRIN2A* (TPM_seal_ = 13) were more highly expressed in seal than in mouse neurons. However, expression values were in the bottom fifth of the expression range of all glutamate receptors in hooded seals (TPM = 1.1–113). Therefore, their higher expression in seals probably does not compensate for the overall reduced expression of glutamate receptors. For comparison, expression values range from TPM = 2.4–210 in mice. Further, a reduced glutamatergic transmission might indicate decreased glutamate pools in hooded seals, which would further reduce energy expenditure because glutamate recycling is costly (Attwell and Laughlin, 2001). Since glutamate also serves as a precursor of GABA (Roberts and Frankel, 1950), a decreased glutamate pool could also lead to the lower GABAergic synaptic transmission that was indicated by our data. This implies that inhibitory effects of GABA release probably do not play the same role in relation to metabolic depression in the seal brain as it has been shown to do in other anoxia-tolerant vertebrates (e.g., Nilsson et al., 1991; Lutz et al., 2003).

### High Capacity for Aerobic Metabolism in Hooded Seal Neurons

Glucose, the key substrate for ATP production, is catabolized in a series of processes: glycolysis, tricarboxylic acid (TCA) cycle and oxidative phosphorylation. Glycolysis provides only 2 ATP but can take place in the absence of oxygen. The TCA cycle and oxidative phosphorylation, on the other hand, produce considerably more energy (38 ATP), but require aerobic conditions. Since marine mammals regularly face limited oxygen availability while diving, many tissues and organs display adaptations that favor anaerobic metabolism (Scholander, 1940; Hochachka and Murphy, 1979; Blix, 2018). However, our data indicate an enhanced capacity for aerobic metabolism in hooded seal neurons compared to the mouse. Genes related to mitochondrial organization and function were more highly expressed in the seal and among the top 10 genes with the highest-fold changes were four components of the electron transport chain. Further, the majority of genes in the GO terms “oxidative phosphorylation” and “respiratory electron transport chain” were significantly more highly expressed in seal than in mouse neurons.

Similar to seal neurons, whale brains (i.e., neurons and glia cells combined) showed a higher expression of the majority of genes in these two GO terms compared to cattle. However, whale genes were not enriched for components of the electron transport chain, possibly because we only had 172 more highly expressed genes in whales that matched our cutoff criteria for the Panther overrepresentation test. In a previous study, Krüger et al. (2020) compared transcriptomes of whales and cattle brain samples, some of which were also used in the present study. We here supplemented that material with additional long-finned pilot whale brain samples. These data confirm and support the conclusions of Krüger et al. (2020); that whale transcriptomes are enriched for the GO terms “oxidative phosphorylation” and “respiratory electron transport chain” (Krüger et al., 2020). These results are, however, partly in contrast to a previous study from our lab that differed from the present study, first by using whole-tissue transcriptomes, instead of neural one’s, from the visual cortex of hooded seals, and, secondly by using the ferret (*Mustela putorius furo*), instead of the mouse, for comparison (Fabrizius et al., 2016). The results presented by Fabrizius et al. (2016) indicate that the GO terms, “oxidative phosphorylation” and “respiratory electron transport chain” were enriched in the ferret, when the category “biological process complete” was considered in PANTHER. However, none of the genes relevant to aerobic metabolism were among the strongest differentially expressed genes in the ferret, other oxidative pathways, e.g., beta oxidation, were not present in the data, and genes indicating general mitochondrial activity or density were not considered – all of which makes a direct comparison of the two studies difficult. The observed difference to the present study may be linked to the choice of non-diving animal model: Mustelids, like the ferret, maintain particularly high (basal) metabolic rates compared to other mammals of similar size (e.g., Casey and Casey, 1979; Worthen and Kilgore, 1981; Korhonen et al., 1983; Harlow, 1994) (although not all studies support this notion (e.g., Brown and Lasiewski, 1972; Balharry, 1994; Harrington et al., 2003), which may explain the discrepancy in results between the present, mouse-based comparison and the ferret-based one of Fabrizius et al. (2016).

Additional to the higher expression of mitochondrial processes, we found an increased expression of genes of the term “mitochondrial envelope” in seal neurons and whale brains, which might be carefully interpreted to signify an enhanced mitochondrial volume density (MVD) compared to in mouse neurons and cattle brains. MVD, commonly studied in muscle and defined as the percentage of muscle fiber volume occupied by mitochondria, can be increased by either increasing the mitochondrial volume or the density of mitochondria. Our data do not allow us to draw any conclusion on whether the observed expression differences indeed reflect these two alternatives, and to the best of our knowledge, data on the MVD of brain tissue of marine mammals have not been published. But the results do imply that hooded seal neurons are adequately endowed with mitochondria, which is interesting given that Mitz et al. (2009) concluded that mitochondrial representation in seal neurons might be poor compared to in astrocytes, based on the distribution of cytochrome c and neuroglobin in the two cell types. To resolve this issue, a direct assessment of mitochondrial densities in different cell types of the seal brain is required. In any case, an increased neuronal mitochondrial density – if confirmed – and their uniform intracellular distribution, would improve the oxygen sink and also reduce its diffusion distance, thereby promoting the cellular uptake of oxygen and allowing a more efficient energy conversion, similar to that observed in diving mammal skeletal muscle (Kanatous et al., 1999; Davis, 2014). Taken together, our data suggest that the hooded seal, and likely other marine mammals, may have high cerebral MVD and high capacity for efficient cerebral aerobic metabolism that enable them to rapidly restore tissue ion and energy homeostasis as oxygen again becomes readily available when they surface after a dive.

### Higher Antioxidant Defense in the Hooded Seal

Since oxidative phosphorylation is one of the major sources of ROS, an increased aerobic metabolism in hooded seal neurons may lead to a harmful exposure to ROS, especially during reoxygenation. Repeated exposure to diving ischemia and reperfusion can increase the production of oxygen radicals and consequently lead to oxidative stress in seals (Elsner et al., 1998). However, generally tissues of seals do not show an increased oxidative damage compared to terrestrial mammals (Vázquez-Medina et al., 2007). For this reason, we have explored the antioxidant defense of hooded seal neurons. We found that the majority of genes in the GO term “antioxidant defense” were more highly expressed in hooded seal neurons (Figure 7). Well-known antioxidants, such as the glutathione-*s*-transferase omega 1 (*GSTO1*, superoxide dismutase 1 (*SOD1*) and glutathione peroxidase 3 (*GPX3*), were among the top 4 antioxidants with the highest-fold changes. Our results go along with the documented elevated antioxidant capacity in several tissues, e.g., blood, lung, heart and liver, in diving compared to terrestrial mammals (Filho et al., 2002; Zenteno-Savın et al., 2002; Vázquez-Medina et al., 2006; García-Castañeda et al., 2017), whereby deeper diving species appear to have a greater antioxidant capacity than shallow divers (Righetti et al., 2014). Further, whale brains showed an elevated expression of *SOD1*, *PRDX6* and *GSTP6*, and genes were enriched for the Reactome pathway “detoxification of reactive oxygen species,” compared to cattle (Krüger et al., 2020). Not every tissue previously studied was found to possess a higher antioxidant defense in marine mammals. Instead, it has been suggested that it depends on the specific tissue function, intensity of aerobic metabolism and the perfusion maintenance during a dive (Vázquez-Medina et al., 2006). Since the functionality of the brain is essential for the integrity of the organism, it appears especially important that the brain is protected from ROS by an improved antioxidant defense system.

### Glycolysis

The brain mainly relies on the aerobic catabolism of glucose for ATP production. A shortage of oxygen can temporarily be overcome via glycolysis, the anaerobic oxidation of glucose providing limited energy (2ATP). When regularly facing limited oxygen availability during dives, it might be beneficial for hooded seals to have an increased anaerobic capacity (Larson et al., 2014). Lactate, either produced by glycolysis or supplied by blood, can also serve as an energy substrate for the brain (Zielke et al., 2009; Czech-Damal et al., 2014), provided oxygen is available. However, accumulation of lactate decreases pH and leads to cell damage (Xiang et al., 2004; Gilbert et al., 2006). We found no clear changes in expression levels of glycolytic genes between hooded seals and mice. While some glycolytic genes were more highly expressed in seal neurons, genes considered as rate-limiting enzymes of glycolysis (Berg et al., 2012), such as hexokinase (*HK1*) and phosphofructokinase (*PFKL* and *PFKP*), were less expressed in seal neurons. Lactate dehydrogenases (*LDHA* and *LDHB*) that also serve as measure for anaerobic capacity (Berg et al., 2012; Davis, 2014) showed no significantly different expression. The glucose transporter 3 (*SLC1A3* or *GLUT3*), present in neurons and facilitating the uptake of glucose and other monosaccharides, was less expressed in seal than in mouse neurons. Alpha glucosidase (*GAA*), essential in the degradation of glycogen to glucose in lysosomes, was among the 10 genes with the lowest expression in seal neurons. In summary, our data imply a slightly lower anaerobic capacity in hooded seal neurons than in mouse neurons. Our results are in line with previous studies that also found no elevated glycolytic capacity in the hooded seal. Similar anaerobic capacities were reported from hooded seal and ferret cortex that were indicated by similar mRNA and protein levels of *LDHA* and *LDHB*. Interestingly LDH was mainly located in fibrillary astrocytes, which implies that glial cells may have a lactate-clearing function in the seal (Hoff et al., 2016). LDH activities were also reported to be similar in brain, muscles and other organs in several diving and terrestrial mammals (Castellini et al., 1981), although previous studies have concluded differently (Messelt and Blix, 1976; Shoubridge et al., 1976; Murphy et al., 1980). However, when harbor and Weddell seals were compared to the dog, muscle LDH activities were 1.4 – 2-fold elevated in both seal species (Kanatous et al., 2002; Polasek et al., 2006).

Since our data represent resting (constitutional) levels, they do not provide information on potential changes in energy metabolism during extended dives. Studies in rat and mice and their derived cell cultures led to controversies as to whether neurons are able to change their glycolytic rate (Dienel, 2019 for a review). However, species adapted to hypoxia were not studied in that respect. Hooded seal neurons may switch to glycolysis in the face of hypoxia during long dives. Hoff et al. (2017) exposed fresh cortical brain slices of the hooded seal to normoxia, hypoxia and hypoxia/reoxygenation *in vitro* to mimic the ‘diving brain’ and found an upregulation of some glycolytic genes compared to normoxia. In our data, the monocarboxylate transporter 4 (*SLC16A3* or *MCT4*) that transports lactate out of the cell, was more highly expressed in the hooded seal neurons. A high removal of lactate might be advantages in long dives when oxygen becomes scarce and when neurons may either switch to glycolysis, or the TCA cycle and malate-aspartate shuttle drop below the glycolytic rate, leading to increased lactate production. This is also compatible with the hypotheses put forward by Mitz et al. (2009), who argued that anaerobic ATP production in seal neurons would require export of lactate, possibly to neighboring astrocytes which appear to be well-equipped for efficient aerobic metabolism of lactate, as also indicated by their high content of *LDHB* (Hoff et al., 2016).

We could not confirm our findings in whales. Two of the rate-limiting enzymes of glycolysis, *PFKL* and *PFKP*, and another 2 glycolytic genes, *ALDOA and PGAM1*, were more highly expressed in whales, Consequently, the brain of whales appear to have an increased glycolytic rate compared to cattle, which is in accordance with previous findings (Shoubridge et al., 1976). In contrast to neurons, as analyzed for the hooded seal, astrocytes are capable of adjusting their glycolytic rate (Dienel, 2019 for a review). Since whole tissue transcriptomes have been studied from whales, it is possible that the higher expression results from cells other than mature neurons, making the study of seal astrocytes a compelling future task. Beyond that it is possible that pinnipeds and whales may have undergone different adaptations regarding their neuronal glycolytic rate.

#### Positive Selection

Using two methods to infer positive selection, we found that *CACNG3*, which regulates the trafficking and channel gating of AMPA glutamate receptors, experienced positive selection in at least one branch (hooded seal). Since *CACNG3* also belongs to the bottom 10 genes with the lowest expression in hooded seals compared to mice, this result supports our suggestion that adaptation of the glutamate-glutamine cycle is important for the hypoxia tolerance of the brain. Further, *CHGA*, which plays a role in neuroendocrine secretion, *BLRVB*, a broad specificity oxidoreductase that is also involved in heme metabolism, and *CAMTA2*, which is possibly involved in cardiac growth and tumor suppression, were also all positively selected in pinnipeds but not in cetaceans. For these genes, it is more difficult to draw a direct link to hypoxia tolerance and to the best of our knowledge, these genes have not been found (or studied) to be positively selected in other hypoxia-tolerant species. In cetaceans, NSF, which is important in the intracellular vesicle transport and PITPNA, involved in phospholipase c signaling, were both positively selected. Again, this supports that signal transduction could be one of the key points in the adaptation to hypoxia.

#### Convergent Evolution in Pinnipeds and Cetaceans

Marine mammals share many phenotypic characteristics that are generally considered as examples of convergent evolution. We found similar differences in expression profiles indicative of reduced signal transmission, of elevated capacity for aerobic metabolism and of increased antioxidant defense in both hooded seals and whales, when compared to mice and cattle, respectively. These findings were also confirmed when hooded seals were compared with cattle. However, when testing genes with the strongest differential expression for positive selection, we found no amino acid substitutions shared between pinnipeds and cetaceans. Positive selection was not a major focus of this study and the here tested subset of genes might not be overall informative. Evidence from other studies suggest that single genes regulating energy homeostasis were positively selected in pinnipeds and cetaceans, at least in some suborders and families (Yu et al., 2011; Wang et al., 2015). More comprehensive studies on positive selection have found evidence for convergent evolution in marine mammals, including two genes involved in antioxidant defense (Foote et al., 2015).

### Expression of Neuroglobin, S100B and Other Genes of Interest

#### Neuroglobin

Neuroglobin is a respiratory protein expressed in the central and peripheral nervous system. Although its function is not fully resolved, its structure and high affinity for oxygen suggest that it may improve oxygen availability to the brain (Burmester et al., 2000). The function of neuroglobin was studied in various neuronal cell lines. While some studies found a neuroprotective effect of neuroglobin overexpression when cells were exposed to hypoxia (Sun et al., 2001; Liu et al., 2009) others did not observe this correlation (Mitz et al., unpublished, but see Burmester and Hankeln, 2009). We found similar expression levels in neurons of hooded seals and mice. Similar neuroglobin protein levels were also found in the cortex of hooded seals, mice and rats (Mitz et al., 2009). However, immunofluorescence revealed that neuroglobin was mainly located in fibrillary astrocytes in the hooded seal brain, whereas it was predominantly found in neurons in mouse and rat brains (Mitz et al., 2009). This suggests that neuroglobin does not contribute to hypoxia tolerance of seal neurons by improving the oxygen availability to these cells as such, although it may give indirect benefits, as discussed by Mitz et al. (2009). Neuroglobin is, however, more highly expressed in the cetacean brain, implying a slightly different role here (Schneuer et al., 2012).

#### S100B

S100B, a calcium binding protein, is involved in many physiological processes such as protein degradation and calcium homeostasis (Donato et al., 2009). S100B was among the 10 genes with the highest expression in hooded seal neurons compared to mouse neurons (FC = 82). Our results confirm a previous comparative transcriptomic study from our group, showing that S100B was 38-fold more highly expressed in the visual cortex of the hooded seal when compared with the ferret (Fabrizius et al., 2016). Further, overexpression of the hooded seal S100B had neuroprotective effects on neuronal mouse cells when exposed to hypoxia and oxidative stress. Reduced ROS-levels and lipid peroxidation suggest that the neuroprotective effect of S100B may be conferred by reducing oxidative stress (Geßner et al., 2020), possibly by limiting accumulation of free calcium and its downstream negative effects, as suggested by Geiseler et al. (2016).

## Conclusion

The marked differences in the transcriptomes of hooded seal and mouse neurons center around a reduced signal transmission, an elevated capacity for aerobic metabolism and an increased antioxidant defense, which we also observed in whale brains when compared to cattle brains. These observations point to an energy budget aimed at reducing ATP expenditure and promoting ATP production. The elevated cerebral aerobic capacity indicated by the present and a previous similar study using whale brains (Krüger et al., 2020) suggests that the brain of diving mammals is designed to efficiently use the oxygen delivered by the blood. This ability could be beneficial to efficiently use oxygen once this becomes readily available during surfacing between dives. Further, the higher expression of components of the mitochondrial envelope is suggestive of higher mitochondrial densities that (i) reduce the diffusion distance for oxygen and (ii) might function as a sink for oxygen, creating an oxygen gradient from the blood and, thus, supporting an efficient use of the elevated blood oxygen stores.

Neurons and astrocytes have a fascinating interplay in neurotransmission and energy metabolism (Bélanger et al., 2011 for a review). Of particular interest is the well characterized ability of astrocytes to remove neurotransmitters from the synaptic cleft. Thus, the here observed adaptations in energy metabolism and neurotransmission of hooded seal neurons likely go hand in hand with corresponding adaptations in astrocytes, making studies of these cells in marine mammals a compelling future task.

Some samples from whales analyzed in this study were not exclusively from the visual cortex and thus, our findings might not be specific to the visual cortex. Nevertheless, it remains speculative whether our observations can be expanded to all other brain regions. In fact, brain imaging methods such as positron emission tomography that identify active brain regions make use of the differential local blood flow and the accompanied glucose utilization (Bélanger et al., 2011). Such task-dependent changes in the activity of local brain regions could also exist in specialist divers, not least during diving (Ramirez et al., 2007). Thus, while the cortex may be of essential relevance, other regions may be less needed during this task. Consequently, blood flow and the accompanied adaptations may well differ between brain regions.

## Data Availability Statement

The datasets presented in this study can be found in online repositories. The names of the repository/repositories and accession number(s) can be found below: https://www.ncbi. nlm.nih.gov/sra/?term=PRJNA785765.

## Ethics Statement

The animal study was reviewed and approved by the Norwegian Animal Welfare Act and with approvals from the National Animal Research Authority of Norway (permits no. 5399 and 7247). Sampling of the long-finned pilot whales was approved by the Convention on Biological Diversity (CBD) and Convention on International Trade in Endangered Species of Wild Fauna and Flora (CITES) (Permit number: E-01456/17).

## Author Contributions

LF and TB conceived the research idea. TB received the funding. CG derived the experimental procedure and performed the experiments and carried out the hooded seal data analysis and wrote the manuscript with input from all authors. WF assisted with the laser capture microdissection. AK analyzed the whale data. LF sampled hooded seal brain. BM sampled the long-finned pilot whales. All the authors contributed to the article and approved the submitted version.

## Conflict of Interest

The authors declare that the research was conducted in the absence of any commercial or financial relationships that could be construed as a potential conflict of interest.

## Publisher’s Note

All claims expressed in this article are solely those of the authors and do not necessarily represent those of their affiliated organizations, or those of the publisher, the editors and the reviewers. Any product that may be evaluated in this article, or claim that may be made by its manufacturer, is not guaranteed or endorsed by the publisher.

## Footnotes

1
http://timetree.org/

2
http://geneontology.org/

3
https://datamonkey.org/
