# Supplementary material for: Transcriptomes Suggest That Pinniped and Cetacean Brains Have a High Capacity for Aerobic Metabolism While Reducing Energy-Intensive Processes Such as Synaptic Transmission
Source: Front Mol Neurosci. 2022 May 9;15:877349. doi: 10.3389/fnmol.2022.877349 (PMC9126210; doi:10.3389/fnmol.2022.877349)
Supplement: Supplementary file 1 [file Data_Sheet_1.docx]

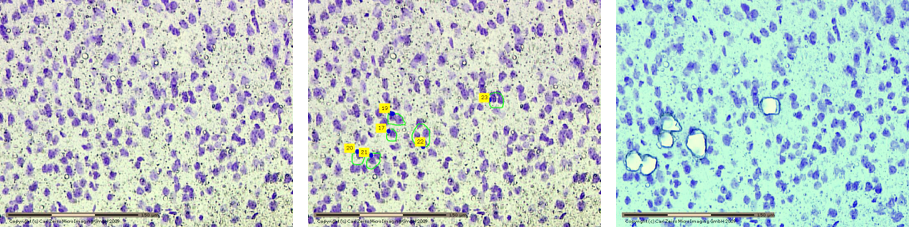


**Suppl. Figure 1:** Neurons of the hooded seal and mouse (latter presented here) visual cortex were excised using Laser Capture Microdessection (LCM) after being stained with cresyl violet acetat. Two to three neurons were circled, cut and catapulted into adhesive caps.

**Suppl. Figure 2:** Enriched GO-slim terms in the category “biological processes” in the neurons of the visual cortex in hooded seals compared to cattle (neurons & glia). Genes used for analysis were significantly higher expressed in hooded seals (fold change ≥ 2). The number of hooded seal genes in every GO-slim term and the FDR-corrected p-value are presented.

**Suppl. Figure 3:** Enriched GO-slim terms in the category “biological processes” in the neurons of the visual cortex of hooded seals compared to cattle (neurons & glia). Genes used for analysis were significantly lower expressed in hooded seals (fold change ≤ -2). The number of hooded seal genes in every GO-slim term and the FDR-corrected p-value are presented.

**Suppl. Figure 4:** Differentially expressed genes in hooded seals (neurons) compared to cattle (neurons & glia). The majority of genes related to neuronal signaling show a lower expression in hooded seals. Only genes with TPM values ≥ 1 and FC ≥ 2 or ≤ -2 were considered.

**Suppl. Figure 5:** Differential expression of genes playing key roles in glutamatergic synaptic transmission and the glutamine-glutamate-cycle in hooded seals (neurons) compared to cattle (neurons & glia). Positive fold changes represent a higher expression in seal neurons.

**Suppl. Figure 6:** Total number of genes related to aerobic metabolism with a higher (red) and lower expression (blue) in the hooded seal (neurons) compared to cattle (neurons & glia).

**Suppl. Figure 7:** Antioxidant genes differentially expressed in hooded seals (neurons) compared to cattle (neurons & glia) visual cortex. Positive fold changes represent a higher expression in hooded seal cortical neurons.

**Suppl. Table 1:** Trimming and RNA-seq results of hooded seals (*Cystophora cristata*, Ccr) and mice (*Mus musculus*, Mmu) reads mapped against the human genome (GRCh38.p13) using the CLC workbench v. 11.0.1. Presented are the mapped and unmapped reads (%), the adapters (%) and the distance of paired reads (paired-distance, bp).

| Sample | SRA accession | # Raw reads | # Trimmed reads | % Mapped | % Adapters | Paired distance (bp) |
| --- | --- | --- | --- | --- | --- | --- |
| *Ccr 1* | SRR17110294 | 70,546,180 | 43,559,930 | 65 | 7 | 26-422 |
| *Ccr 2* | SRR17110293 | 73,882,938 | 55,124,622 | 65 | 5 | 30-484 |
| *Ccr 3* | SRR17110292 | 80,438,260 | 57,702,171 | 66 | 5 | 32-452 |
| **mean** |  | **72,445,103** | **53,002,681** | **65** | **6** | **29-455** |
| *Mmu 1* | SRR17110291 | 26,215,027 | 52670835 | 52 | 5 | 18-483 |
| *Mmu 2* | SRR17110290 | 28,321,624 | 57451981 | 52 | 4 | 12-596 |
| *Mmu 3* | SRR17110289 | 26,701,034 | 55516980 | 50 | 4 | 12-628 |
| **mean** |  | **27,079,228** | **55,213,265** | **51** | **4** | **14-569** |

**Suppl. Table 2:** Summary of illumina sequencing. The number of reads per sample before and after quality trimming is given. Samples newly sequenced in this study are printed in bold. The percentage of reads mapped to the human genome is denoted. Gme VC = Globicephala melas (visual cortex); Gme CE = Globicephala melas (cerebellum); Oor = Orcinus orca; Bac = Balaenoptera acutorostrata; Bmy = Balaena mysticetus; Bta 1 = Bos Taurus sample 1; Bta cowb = Bos taurus sample b; Bta cowc = Bos taurus sample c; Bta br1 = Bos taurus sample br1, br2 = Bos taurus sample br2

| Sample | SRA accession | Raw reads | Trimmed | Mapped reads % |
| --- | --- | --- | --- | --- |
| Gme VC1 | SRR8305674 | 49,465,538 | 49,462,293 | 53.83 |
| Gme CE1 | SRR8305675 | 41,444,974 | 41,442,287 | 49.6 |
| **Gme VC2** | SRR17090398 | 61,089,378 | 61,080,507 | 56.22 |
| **Gme CE2** | SRR17090399 | 58,640,018 | 58,631,402 | 52.79 |
| **Gme VC3** | SRR17090396 | 57,407,262 | 57,398,781 | 56.5 |
| **Gme CE3** | SRR17090397 | 87,256,932 | 87,254,523 | 53.45 |
| Oor | SRR8305677 | 29,964,586 | 29,861,576 | 47.37 |
| Bac | SRR918699 | 51,470,260 | 51,341,266 | 52.02 |
| Bmy | SRR1685414 | 21,182,210 | 17,750,937 | 47.96 |
| Bta1 | SRR8305676 | 32,089,626 | 32,086,347 | 47.4 |
| Bta cowb | SRR594482 | 56,890,388 | 56,292,520 | 62.84 |
| Bta cowc | SRR594491 | 67,256,226 | 64,242,921 | 63.72 |
| Bta br1 | SRR636934 | 53,310,044 | 53,119,486 | 54.21 |
| Bta br2 | SRR636935 | 29,597,408 | 29,429,907 | 55.67 |

**Suppl. Table 3:** Genes involved in the regulation of glutamatergic synaptic transmission. Negative fold changes present a lower expression in seal compared to mouse neurons.

| **Gene symbol** | **Fold change** | **Name** |
| --- | --- | --- |
|  |  |  |
| ***negative regulation of synaptic transmission, glutamatergic (GO:0051967)*** | | |
| Adora1 | -3.2 | adenosine A1 receptor |
| Htr2a | 3.66 | 5-hydroxytryptamine (serotonin) receptor 2A |
| Pla2g6 | 2.02 | phospholipase A2, group VI |
|  |  |  |
| ***positive regulation of synaptic transmission, glutamatergic (GO:0051968)*** | | |
| Cacng3 | -19.2 | calcium channel, voltage-dependent, gamma subunit 3 |
| Cckbr | -19.5 | cholecystokinin B receptor |
| Glul | 3.3 | glutamine synthetase |
| Gria4 | -3.15 | glutamate receptor, ionotropic, AMPA4 (alpha 4) |
| Iqsec2 | -2.94 | IQ motif and Sec7 domain 2 |
| Nlgn1 | -4.9 | neuroligin 1 |
| Ptk2b | -3.01 | PTK2 protein tyrosine kinase 2 beta |
| Reln | -3.41 | reelin |

**Suppl. Table 4:** Expression of genes involved in glycolysis in the mouse and hooded seal cortical neurons when transcripts were mapped to the mouse reference genome. Negative fold changes indicate a lower expression in seal neurons compared to the mouse. Rate-limiting enzymes are in bold letters.

| **Gene symbol** | **Gene name** | **Fold change** | **FDR** | **mouse TPM** | **seal TPM** |
| --- | --- | --- | --- | --- | --- |
|  |  |  |  |  |  |
| ***Part 1: glucose to fructose-1,6-bisphosphate*** | |  |  |  |  |
| **HK1** | hexokinase 1 | -1.3 | 0.797 | 2106 | 422 |
| **PFKL** | phosphofructokinase, liver | -5.1 | 0 | 283 | 58 |
| **PFKM** | phosphofructokinase, muscle | 2.7 | < 0.001 | 5930 | 1180 |
| PFKP | phosphofructokinase, platelet | -1.6 | 0.018 | 1639 | 326 |
| ALDOA | aldolase A | -1.6 | 0.008 | 9722 | 1946 |
| TPI1 | triosephosphatisomerase | 1.8 | < 0.001 | 9726 | 1941 |
|  |  |  |  |  |  |
| ***Part II: glyceraldehyde-3-phosphate to pyruvate*** | | |  |  |  |
| PGK1 | phosphoglycerate kinase 1 | 3.1 | < 0.001 | 7931 | 1582 |
| PGAM1 | phosphoglycerate mutase 1 | - | - | - | - |
| PGAM2 | phosphoglycerate mutase 2 | 2.5 | 0.000 | 62 | 12 |
| ENO2 | enolase 2 | -73.0 | < 0.001 | 77 | 15 |
